# Unraveling the genetic architecture of subtropical maize (*Zea mays* L.) lines to assess their utility in breeding programs

**DOI:** 10.1186/1471-2164-14-877

**Published:** 2013-12-13

**Authors:** Nepolean Thirunavukkarasu, Firoz Hossain, Kaliyugam Shiriga, Swati Mittal, Kanika Arora, Abhishek Rathore, Sweta Mohan, Trushar Shah, Rinku Sharma, Pottekatt Mohanlal Namratha, Amitha SV Mithra, Trilochan Mohapatra, Hari Shankar Gupta

**Affiliations:** 1 Division of Genetics, Indian Agricultural Research Institute, Pusa, New Delhi 110012, India; 2 International Crops Research Institute for the Semi-Arid Tropics, Patancheru, 502324, Andhra Pradesh, India; 3 National Research Centre on Plant Biotechnology, Pusa, New Delhi 110012, India

**Keywords:** Subtropical maize, Genome-wide SNPs, Linkage disequilibrium, Population structure, Association mapping, Genetic diversity

## Abstract

**Background:**

Maize is an increasingly important food crop in southeast Asia. The elucidation of its genetic architecture, accomplished by exploring quantitative trait loci and useful alleles in various lines across numerous breeding programs, is therefore of great interest. The present study aimed to characterize subtropical maize lines using high-quality SNPs distributed throughout the genome.

**Results:**

We genotyped a panel of 240 subtropical elite maize inbred lines and carried out linkage disequilibrium, genetic diversity, population structure, and principal component analyses on the generated SNP data. The mean SNP distance across the genome was 70 Kb. The genome had both high and low linkage disequilibrium (LD) regions; the latter were dominant in areas near the gene-rich telomeric portions where recombination is frequent. A total of 252 haplotype blocks, ranging in size from 1 to 15.8 Mb, were identified. Slow LD decay (200–300 Kb) at *r*^*2*^ ≤ 0.1 across all chromosomes explained the selection of favorable traits around low LD regions in different breeding programs. The association mapping panel was characterized by strong population substructure. Genotypes were grouped into three distinct clusters with a mean genetic dissimilarity coefficient of 0.36.

**Conclusions:**

The genotyped panel of subtropical maize lines characterized in this study should be useful for association mapping of agronomically important genes. The dissimilarity uncovered among genotypes provides an opportunity to exploit the heterotic potential of subtropical elite maize breeding lines.

## Introduction

Maize (*Zea mays* L.) is one of the most important global food crops, and is of increasing agricultural importance in India [1]. According to USDA estimates (http://www.fas.usda.gov/psdonline/circulars/production.pdf), an area of 8.68 million hectares in India was used to produce 21.6 million tons of maize during 2011–2012. Maize is used in India for various applications, ranging from food and feed to industrial purposes. Although maize ranks third in terms of crop production, demand is expected to double by 2050, given the growth of the Indian population and the preference for maize over other cereals. Currently, maize productivity in India is 2.49 tons per hectare, which is far lower than the global average of 5.2 tons per hectare. This limited output can be explained by production constraints, which range from biotic and abiotic stresses to unexploited heterotic potential. Elucidating the genetic architecture of maize at the molecular level would aid the development of cultivars better suited to meet increasing demands.

Modern maize arose from the domestication of teosinte (*Zea mays* ssp. *parviglumis*), which occurred in southwestern Mexico approximately 9000 years ago [2]. Maize slowly spread across the Americas in numerous forms that were locally adapted to tropical as well as temperate climatic conditions [2]. Although most Asian corn is derived from recently introduced Caribbean-type flints [3], maize lines with primitive features, distinct from Mexican lines, are found in the northeastern Himalayan region [4]. Indian maize races are classified into four groups: primitive, advanced or derived, recently introduced, and hybrids. Despite thousands of years of domestication, maize has retained a great deal of allelic diversity [5]. Maize polymorphisms between two diverse lines are estimated to occur every 44 bp on average [6], a higher SNP frequency than between humans and chimpanzees. Millions of single nucleotide polymorphisms (SNPs) and indels, critical for understanding trait architecture, have been identified in maize using diverse inbred lines [6].

Linkage disequilibrium (LD) is the non-random association of alleles at two or more loci in a population. An understanding of LD patterns in a population is useful for association mapping [7,8]. LD decay, the rate at which LD breaks down, occurs slowly in commercial maize germplasm [9-11]; in numerous other germplasm lines, including landraces, it occurs within a few Kb because of high rates of recombination [12-16]. In maize, extensive LD has been found around *Y1*[14] and in a 1-Mb region on chromosome 10 [17]. Many LD blocks of varying sizes have also been identified by genome-wide screening [6,7,18-20]. Another important consideration during association mapping is population structure. Agronomically important traits are rigorously selected for in breeding programs, establishing population structure in the germplasm. Population structure can cause significant fluctuations in allele frequencies across subpopulations, creating unexpected LD between loci that are actually unlinked [21]. Several methods, such as genomic control [22,23], structured association [24], principal component analysis (PCA) [25], non-metric multidimensional scaling [26], and a unified mixed model approach [27], have been used to minimize the effects of population structure on association mapping.

The study of genetic relationships among breeding lines is essential not only for parental selection, but also for hybrid development and heterotic grouping [28]. Diversity analyses can be performed at morphological, geographical, and functional levels [29-33]. The diversity found among Indian lines is due to the crossing of Indian germplasm with foreign strains, particularly those from the USA [34]. This cross-breeding has resulted in augmented yield and heterosis [35-37]. The initial focus of Indian maize breeding programs was the development of double-cross hybrids using inbred lines, with attention later shifting to early-maturing composites. Over the last two decades, interest has centered around the development of single-cross hybrids, with several hybrids adapted to various Indian agro-climatic conditions released as a result.

A comprehensive knowledge of the genetic architecture of maize populations is useful for exploiting germplasm for various breeding purposes. The present study was carried out to (1) characterize subtropical genotypes adapted to Indian conditions using genome-wide SNPs; (2) elucidate the LD and population structure of the genotype panel for use in association mapping; and (3) assess genotype genetic diversity to develop heterotic parental combinations.

## Methods

### Plant material

A panel of 240 subtropical or tropical genotypes, consisting of inbred lines adapted to subtropical climates and developed at different breeding stations in India or by the International Maize and Wheat Improvement Center (CIMMYT), were used for SNP genotyping. These elite inbreds had putative genes segregating for biotic and abiotic stress tolerances, nutritional traits, and agronomic traits (Additional file 1: Table S1).

### SNP genotyping and assay development

Total genomic DNA was isolated from each of the 240 samples using a Nucleopore DNASure plant mini kit (Genetix Biotech Asia, New Delhi, India). Quantity and quality of isolated DNA samples were checked with a NanoDrop ND-1000 spectrophotometer (Thermo Scientific, Wilmington, DE, USA), followed by validation by 1% agarose gel electrophoresis. SNP detection was performed using the Infinium HD Assay Ultra protocol (Illumina, San Diego, CA, USA). DNA samples (50 ng in 4 μl) were hybridized to a Maize SNP50 BeadChip.

### Data curation

**Figure 1:**
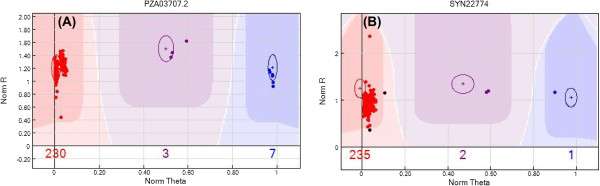
**Clustering pattern of high-quality SNPs analyzed with GenomeStudio.** All SNPs in the 240 genotypes assessed were grouped into three clusters: AA (red), AB (purple), and BB (blue). The normalized θ range was 0–0.2 and 0.8–1 for homozygous clusters AA and BB, respectively. The black datapoints were regarded as “no calls.” **A)** PZA-03707.2 represented high-quality SNPs corresponding to clear homozygous clusters (AA or BB). **B)** Datapoints falling within the range of the normalized θ values were manually adjusted into the respective clusters for SYN22774 to increase the calling accuracy. SNPs with inadequate cluster separation were deleted from the 56,110 SNP set.

GenomeStudio version 2010.3 was used to analyze the SNP genotyping data. GenomeStudio clusters genotype calls into AA, AB, and BB groups that are converted using TOP/BOTTOM rules into different allelic combinations: A/C, A/G, A/T, C/G, C/T, and G/T (in TOP, A/G indicates that allele 1 is A and allele 2 is G, whereas in BOTTOM, A/G indicates that allele 1 is G and allele 2 is A). In this study, every SNP was scored using GenTrain (GT) and Cluster Separation (CS) [38] selection criteria. True-positive signals reflected as background noise were manually assigned to their respective clusters based on a defined normalized θ value (Figure 1). Only reliable SNPs showing distinct cluster separation were retained in the curated set. SNPs not included in any cluster were categorized as “no calls.”

### SNP characteristics

Polymorphism information content (PIC), minor allelic frequency (MAF), and genetic diversity (GD) were calculated using the Genetics package in R [39].

### Population structure

ADMIXTURE version 1.20 [40] was used to study population structure using a subset of 8,278 SNPs having pairwise *r*^2^ values < 0.1 distributed randomly across the genome. A subset was chosen to minimize the effects of LD, as the model employed by this software program does not explicitly take LD into consideration. The “Expectation Maximization” clustering algorithm was used with numerous clusters (K) ranging from 2 to 7. The algorithm was executed five times for each K value. To select the substructure level corresponding to the best partitioning, we also performed five-fold cross-validation.

### Principal component analysis

Principal component analysis was performed using the R package SNPRelate [41]. An LD-based pruned set of SNPs was first created with an LD threshold of 0.2 to avoid the strong influence of SNP clusters. Using the snpgdsPCA function in SNPRelate, PCA was then conducted (MAF ≥ 0.05 and missing rate ≤ 0.15). The percentage of variation explained was calculated for the first 16 principal components, and the first four components were used for plotting the genotypes on a two-dimensional scale.

### Assessment of genetic diversity

A genetic dissimilarity matrix was calculated from 29,619 SNPs using Roger’s modified distance [42] with the ade4 package in R. The dissimilarity values were used for construction of a dendrogram in Darwin 5.0 [43] using the weighted neighbor-joining (NJ) method.

### Linkage disequilibrium

The LD pattern across chromosomes was investigated using TASSEL 3.0.132 [44]. Pairwise LD explained by *r*^*2*^ was determined for 29,619 high-quality SNPs. LD patterns without any MAF threshold and with thresholds of 5% and 10% were examined. Haploview 4.2 [45] was used to assess haplotypes under high LD using three models: confidence interval (CI), four gamete rule (FGR), and solid spine of LD (SS). We incorporated SNPs up to a distance of 10 and 20 Mb to measure haplotype blocks based on pairwise correlations. Increasing the window size enabled us to assess more SNPs comprising haplotype blocks on chromosomes.

## Results

### SNP performance

Each SNP was assigned GT and CS scores across the 240 Infinium-assayed genotypes. Approximately 92.6% of GT scores and 80% of CS scores were in the range of 0.7–0.9 and 0.7–1, respectively (Additional file 2: Figure S1). Selection of the 29,619 high-quality SNPs for data analysis was performed after removal of “no calls” (19%), monomorphs (0.9%), unmapped SNPs (22.2%), SNPs with a MAF < 0.05 (5%), and SNPs showing greater than 5% heterozygosity (2%). When no MAF threshold was applied, 32,444 SNPs remained; the use of MAF thresholds ≥ 5% and ≥ 10% yielded 29,619 and 25,701 SNPs, respectively. The distribution of curated SNPs ranged from 1,317 on chromosome 2, to 3,811 on chromosome 1 (Additional file 3: Figure S2). Inter-marker distances varied from 2 bp on chromosomes 1, 3, 4, 6, 7, 8, and 9, to 2.83 Mb on chromosome 6, with an overall mean across 10 chromosomes of 70 Kb. Chromosome 8, where SNPs occurred on average at 59-Kb intervals, was the most saturated. Mean PIC, MAF, and GD values were 0.35, 0.25, and 0.36, respectively (Additional file 4: Figure S3). In the selected SNP data, 68% of SNPs had PIC values > 0.25, and 69% of SNPs had GD values > 0.29.

### Linkage disequilibrium

**Figure 2:**
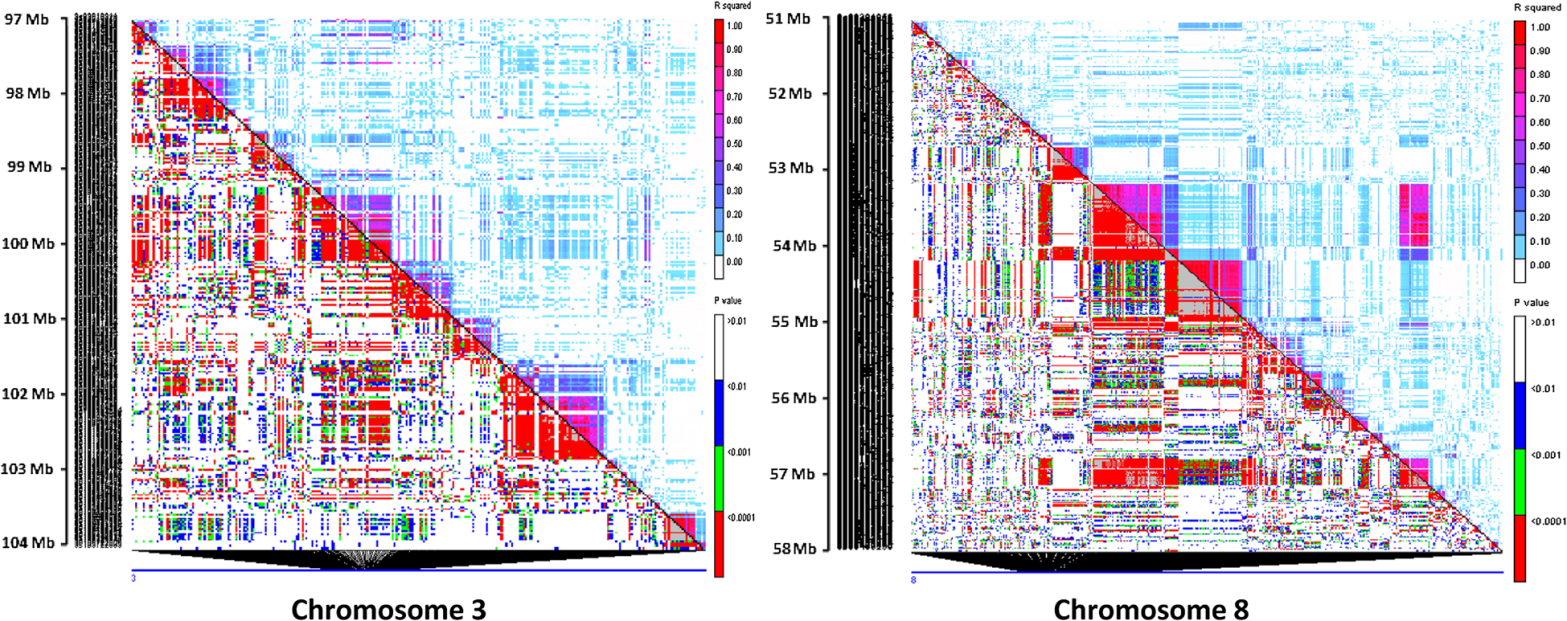
**Heatmaps representing variation in LD on chromosomes 3 and 8.** The markers were aligned on the *x* and *y* axes according to their chromosomal positions. The squared correlation coefficient (*r*^*2*^*)* values are denoted by a color scale from white (0.0) to dark red (1.0) in the upper triangle. The *p* values ranging from non-significant (> 0.01; white) to highly significant (< 0.0001; red) are shown in the lower triangle. The high LD regions occurred between 94.6 and 95.1 Mb on chromosome 3, and 48.7 and 51.7 Mb on chromosome 8.

LD estimation revealed a mean *r*^*2*^ of 0.23 across all chromosomes. Mean *r*^*2*^ was slightly higher on chromosomes 4, 5, and 8 (0.25) compared with chromosome 2 (0.21). Across the entire genome, 3,248 pairwise SNPs were classified as high LD (*r*^*2*^ ≥ 0.8), most of which (13%) were present on chromosome 8. Clusters of SNP pairs in high LD were found on chromosomes 3 and 8 (Figure 2).

Haplotype patterns were analyzed in Haploview with a 20-Mb window under three distinct models: CI, FGR, and SS. A total of 5,158 pairwise SNPs were found to persist in haplotype blocks on all chromosomes. The total number of haplotype blocks ranged from 18 under the CI model to 252 under the FGR model (Additional file 5: Table S2). The latter model suggested a maximum of 74 blocks on chromosome 2, whereas the SS model suggested 68. The maximum average length per block (2,825 Kb) was computed using the SS model. The FGR and SS models identified the largest block on chromosome 3, which was 15.8 Mb and spanned 262 SNPs. The CI model identified the largest block, 4,555 Kb, on chromosome 4, with a coverage of 36 SNPs. The percentage of the chromosome covered by blocks ranged from 0–2% (CI), 5.2–47.4% (FGR), and 2.6–61.2% (SS). The number of blocks varied from one chromosome to another. Chromosome 9 had a minimum of 11 blocks (3–2,787 Kb) and chromosome 2 had a maximum of 74 blocks (1–9,680 Kb), irrespective of the model used. Chromosome 2 also had more than twice the number of blocks as chromosome 4, despite having the lowest total number of SNPs.

When window size was reduced from 20 to 10 Mb, a change in haplotype block patterns was observed under FGR (chromosome 3) and SS (chromosomes 2 and 3) models (Additional file 5: Table S2). The size of the largest block dropped from 15.8 Mb to 7.5 Mb (chromosome 3) under the SS model, which was equivalent to a significant difference of 205 SNPs. Using a 20-Mb window size, average block length ranged from 711.7 Kb (chromosome 9) to 2,825 Kb (chromosome 3). Chromosome 2 had the highest percentage of markers constituting blocks (72.05%). With a 10-Mb window, the average block length ranged from 711.7 Kb (chromosome 9) to 1,919.3 Kb (chromosome 4) under the SS model. The total number of blocks calculated across all chromosomes under the FGR (253) model was equivalent to that of the SS model (252), whereas the CI model estimate comprised only 18 blocks.

### LD decay

**Figure 3:**
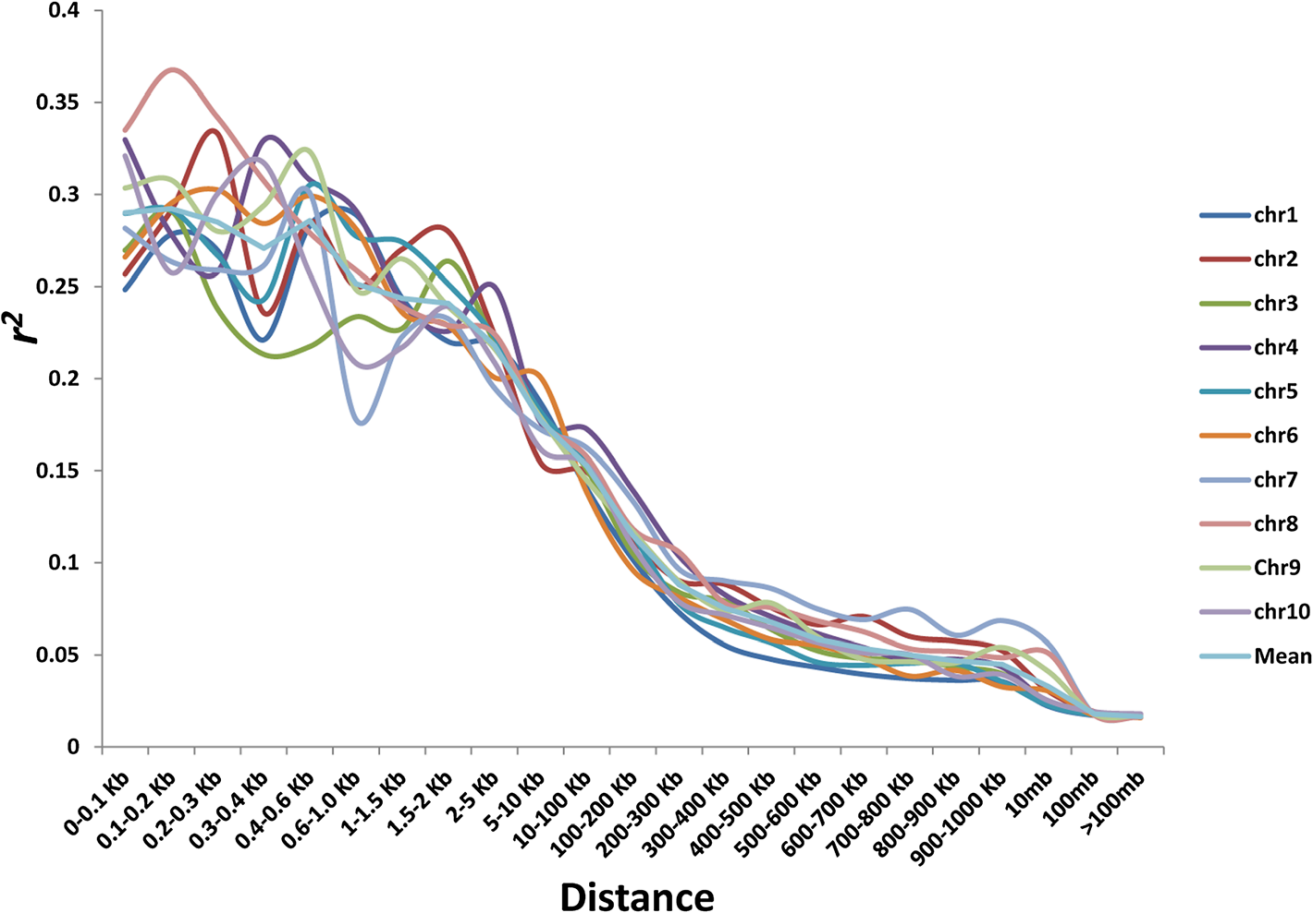
**Linkage disequilibrium (LD) decay pattern of SNPs in all chromosomes.** The mean *r*^*2*^ value was measured across 240 genotypes using SNPs with a MAF of ≥ 0.05. LD decay was considered at both the *r*^*2*^ ≤ 0.1 and *r*^*2*^ ≤ 0.2 levels.

At a mean *r*^*2*^ ≤ 0.1 and with SNPs having a MAF ≥ 5%, LD decayed within 200–300 Kb, although this varied within and across chromosomes (Figure 3). LD decay was most rapid on chromosome 6 (100–200 Kb) and slowest on chromosome 4 (300–400 Kb). On the remaining chromosomes, which covered approximately 82% of the genome, the decay distance was 200–300 Kb (Table 1).

**Table 1:** **The pattern of linkage disequilibrium decay (Kb) at** ***r***^***2***^** ≤ 0.1 and** ***r***^***2***^**≤ 0.2 levels across all chromosomes**

**Chromosome**	**LD decay (Kb)**	
	***r***^***2***^**≤ 0.1**	***r***^***2***^**≤ 0.2**
1	200-300	5-10
2	200-300	5-10
3	200-300	5-10
4	300-400	5-10
5	200-300	5-10
6	100-200	10-100
7	200-300	2-5
8	200-300	5-10
9	200-300	5-10
10	200-300	5-10
**Mean**	**200**-**300**	**5-10**

LD breakdown at a mean *r*^*2*^ ≤ 0.2 occurred on average within 5–10 Kb across the entire maize genome (Figure 3). On chromosomes 6 and 7, the mean decay distance was 10–100 Kb and 2–5 Kb, respectively, whereas it was the same as the global average on the remaining chromosomes. On chromosome 7, LD decay at *r*^*2*^ ≤ 0.2 (2–5 Kb) was found to be more rapid than at *r*^*2*^ ≤ 0.1 (200–300 Kb). On chromosomes 1, 2, 3, 5, 8, 9, and 10, the LD distance dropped from 200–300 Kb at *r*^*2*^ ≤ 0.1 to 5–10 Kb at *r*^*2*^ ≤ 0.2 (Table 1).

The pattern of LD decay was also studied in the absence of a MAF threshold, in which 32,444 SNPs were taken into account, and with a MAF cut-off of 10%, which included 25,701 SNPs. At a mean *r*^*2*^ ≥ 0.1 under all three MAF criteria, LD decayed within 200–300 Kb across the genome. On chromosome 1, LD decayed within 100–200 Kb when no MAF cut-off was applied, and within 200–300 Kb with MAF thresholds ≥ 5% and 10% (Additional file 6: Figure S4). Chromosomes 6 and 8 also showed variable LD decay patterns when SNPs based on a MAF threshold ≥ 10% were used.

### Population stratification

**Figure 4:**
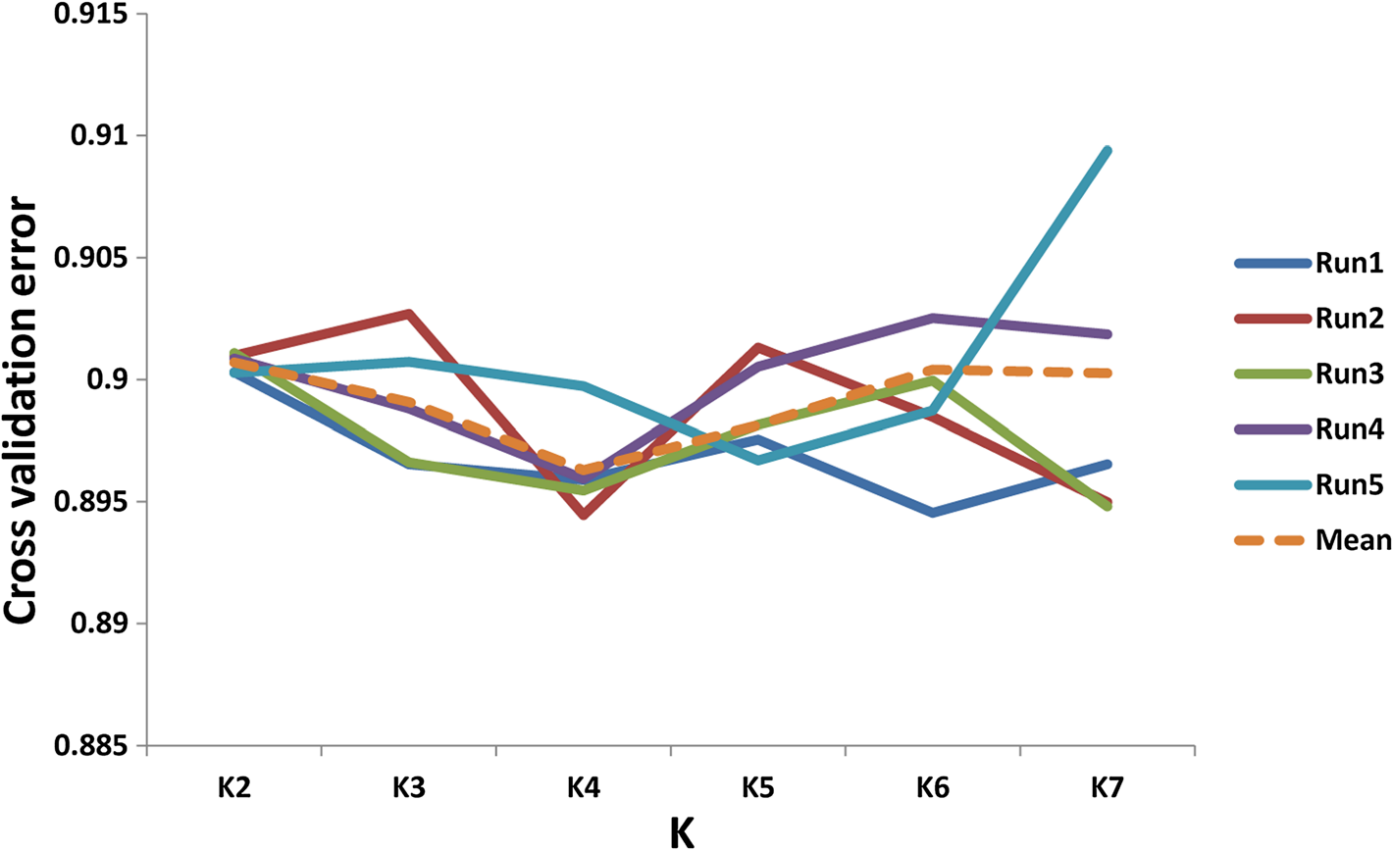
**Cross-validation at different K levels.** The best partition, K = 4, was selected by five-fold cross-validation.

ADMIXTURE with K ranging from 2–7 was used to identify subgroups present in our association mapping panel (Additional file 7: Figure S5). Based on consistent five-fold cross-validation error among runs, K = 4 was selected as the best partition (Figure 4). Admixture results revealed that out of 240 individuals, 18% had a membership value (Q) > 0.8 and were distributed across subgroups (Additional file 8: Table S3).

G1, the largest group, comprised 63% of the genotypes, with G2, G3, and G4 accounting for 27%, 7%, and 3%, respectively (Additional file 8: Table S3). The most distinct maize lines from all maturity groups (early, medium, intermediate, and late) were clustered in G1. The major lines in this group—PANT, BAJIM, CM, and CML—possessed the distinct characteristics of orange-colored grains, acidic soil tolerance, and resistance against ear rot, tar spot, stalk rot, leaf blight, rust, southwestern corn borer, and fall armyworm. This group comprised 39% of yellow lines from different breeding programs at Almora, Amberpet, Bajaura, IARI, Karnal, Ludhiana, Nagenaha, and Udaipur breeding centers. Most CML lines (52) were grouped into G2, which also included BAJIM, BML, CM, CML, DTPW, HKI, HPLET, and V lines. These lines originated from Almora, Amberpet, Bajaura, DMR, IARI, Karnal, and Ludhiana breeding programs. Approximately 63% of the yellow lines drawn from Karnal and Almora were clustered in G3. Equal proportions of yellow lines from these breeding programs were grouped into G4.

**Figure 5:**
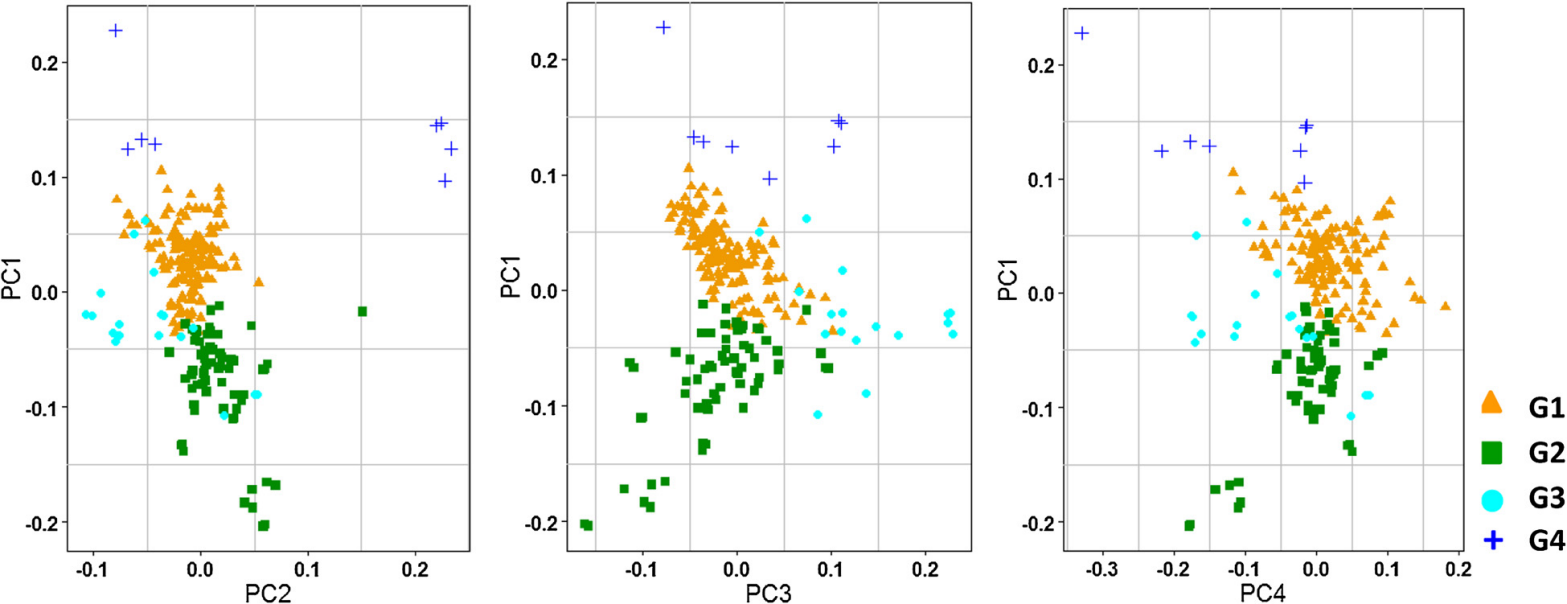
**Principal components explained the distribution pattern of the genotypes.** All genotypes in the PCA plot were color coded as per the ADMIXTURE groupings and showed similar grouping patterns except for the minor groups. Some of the genotypes from the two minor clusters from ADMIXTURE were mixed with the major groups.

Principal components were generated for the SNP datasets. The first component was plotted against the second, third, and fourth components to elucidate genotype grouping patterns. The four components explained 8.2%, 7.3%, 3.9%, and 3.2% of the variation, respectively, and clearly revealed the existence of two major groups in the association mapping panel (Figure 5). Two minor groups were distributed around these two major clusters.

### Genetic diversity

**Figure 6:**
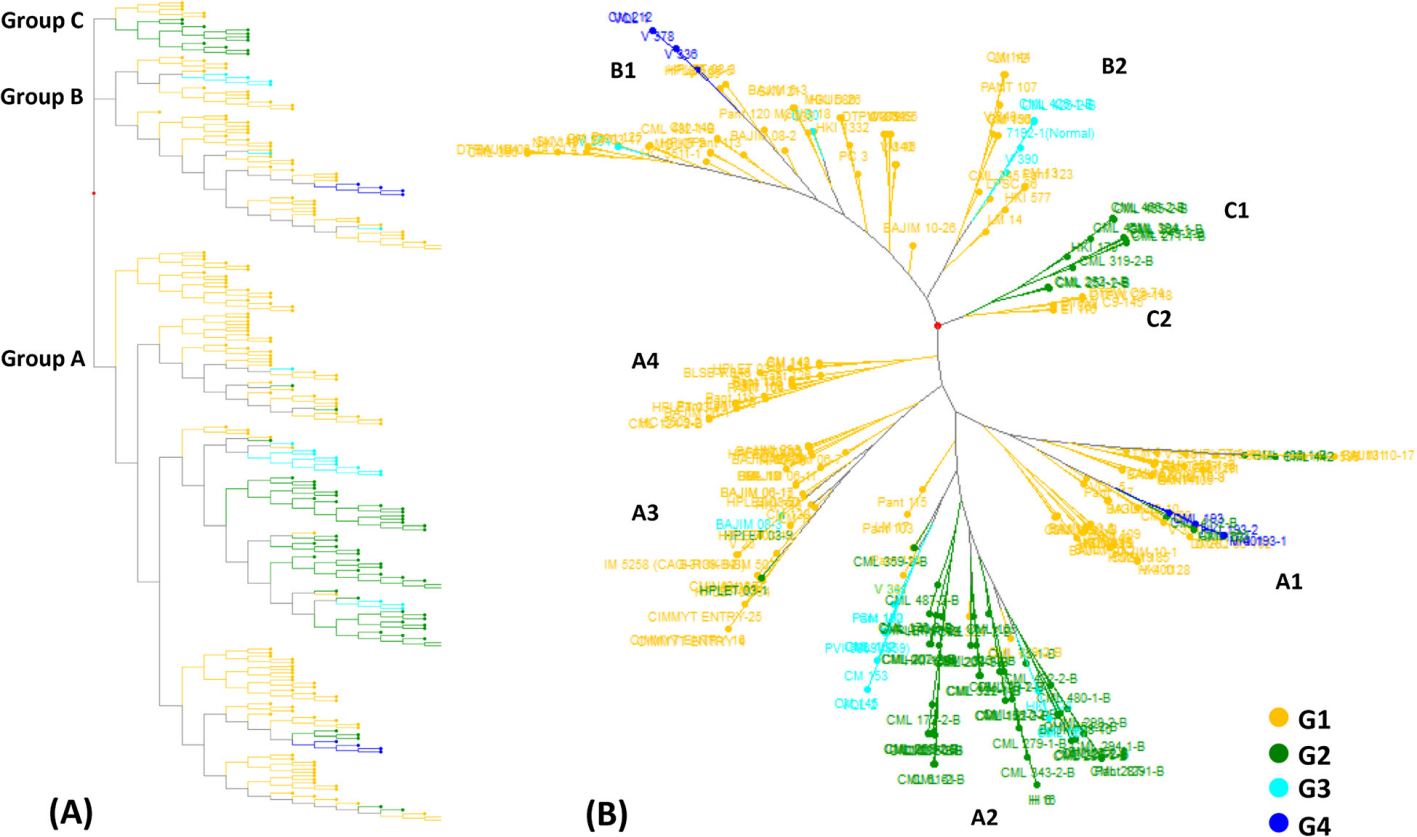
**Clustering of genotypes into three major groups based on genetic dissimilarity.A)** Dendrogram of 240 subtropical maize genotypes based on their genetic distance representing the major groups in a hierarchical topology, **B)** Subgroups are shown in a radial topology. Colors show the different subgroups inferred by ADMIXTURE.

Pairwise genetic dissimilarity coefficients between genotypes varied, with observed values as high as 0.45 and an average of 0.36. Ninety-nine percent of the genotypes had a GD higher than 0.31 (Additional file 9: Figure S6). A dendrogram showing three major groups—A, B, and C, with 4, 2, and 2 subgroups, respectively—was obtained from the genetic dissimilarity matrix (Figure 6).

Group A was the largest group, with 69% of the genotypes, followed by group B with 23% and the remainder in group C. Approximately 53 CML-derived lines (including 25 white lines) constituted the majority of group A, and were characterized by tolerance to acidic soil, lodging, and drought, and resistance against ear rot, tar spot, stalk rot, leaf blight, rust southwestern corn borer and fall armyworm. Yellow lines drawn from Karnal, Almora, and Ludhiana breeding programs constituted the majority of group B. These lines were drought and acid soil tolerant, and resistant to stalk rot and sorghum downy mildew. Group C included 37% of the yellow lines bred at Karnal and Udaipur, of which 63% were CML-derived, one a multiply-resistant genotype (CML 394).

Subgroups A1, A2, A3, and A4 had mean dissimilarity coefficients of 0.37, 0.35, 0.35, and 0.34, respectively. A1 included 39% of A-group genotypes, A2 31%, A3 19%, and A4 11%. In the A1 subgroup, the breakdown of lines was as follows: 2% BAJIM, 5% CM, 64% CML, 5% HKI, 2% HPLET, 8% PANT, and 2% BML. Two of these were drought tolerant, and 41% were white lines. Group B contained two clusters, B1 (72%) and B2 (28%), with mean dissimilarity coefficients of 0.343 and 0.34, respectively. The B1 subpopulation comprised one white line and four drought-tolerant lines. Group C was subdivided into two clusters, C1 (69%) and C2 (31%); these were distinct clusters with genetic distances of 0.3 and 0.34, respectively. Group C1 included one HKI and 10 CML lines.

When the groups uncovered in the ADMIXTURE analysis were compared with those based on genetic distances, group A, the largest group in the genetic dissimilarity dendrogram, contained 59%, 32%, 7%, and 2% of the genotypes from ADMIXTURE groups G1, G2, G3, and G4, respectively. The smallest group, group C, comprised 69% of the lines from G2 and the lines from G1 (at K = 4). Genetically distant lines V338 and CML 442 were included in G1 and G2 (at K = 4) in the ADMIXTURE analysis. Q-values of these genotypes were 0.53 and 0.65, respectively.

## Discussion

### SNP performance

A total of 240 genotypes were screened to identify genome-wide SNPs and to assess population allelic variation. Of 56,110 identified maize SNPs, 98% were detected in this screening, comparable to the number reported in other experiments [13,46]. We used two quality parameters, GT and CS [38], to differentiate genotype clusters as AA (homozygote), AB (heterozygote), and BB (homozygote). Earlier studies revealed high-quality SNPs with CS scores > 0.3 [47] and GT scores > 0.8 [48]. In our study, GT scores ranged from 0.3–0.9 and CS scores from 0.1–1 for the full marker set. Finally, 29,619 high-quality SNPs were obtained after setting GT and CS thresholds ≥ 0.7. Our study identified reliable SNPs and well-defined genotype clusters, as can be seen in the genoplot in Figure 1, reducing the chance of genotyping errors [38,49].

The set of genotypes screened in our study represents the most saturated panel to date of subtropical maize lines adapted to the Indian climate. As reported in other studies, tropical lines have more rare SNPs than temperate lines [13]. In our panel, one SNP was detected every 70 Kb, and thus 29,619 SNPs were useful for assessing the genetic architecture of the subtropical lines. The SNP density for specific genes was 43–623 bp in the study by Jones et al. (2009) [50] and 41–130 bp in that of Ching et al. (2002) [51]. In the present study, SNPs genotyped on chromosome 8 covered the maximum genomic area at an average interval of 59 Kb. Several genomic regions encompassing large distances had no SNPs, including a 2.2-Mb region on chromosome 1, a 2.22-Mb region on chromosome 9, and a 2.83-Mb region on chromosome 6. The latter region was also found in the B73 genome [46]. Approximately 8,963 SNPs with high GD and PIC values were detected with a MAF of 0.4 in this subtropical panel. The highest PIC and GD values were equivalent to those observed in tropical and temperate lines [12,31]. The mean PIC value was quite close to that computed for Chinese and American lines [52].

### LD and LD decay

**Figure 7:**
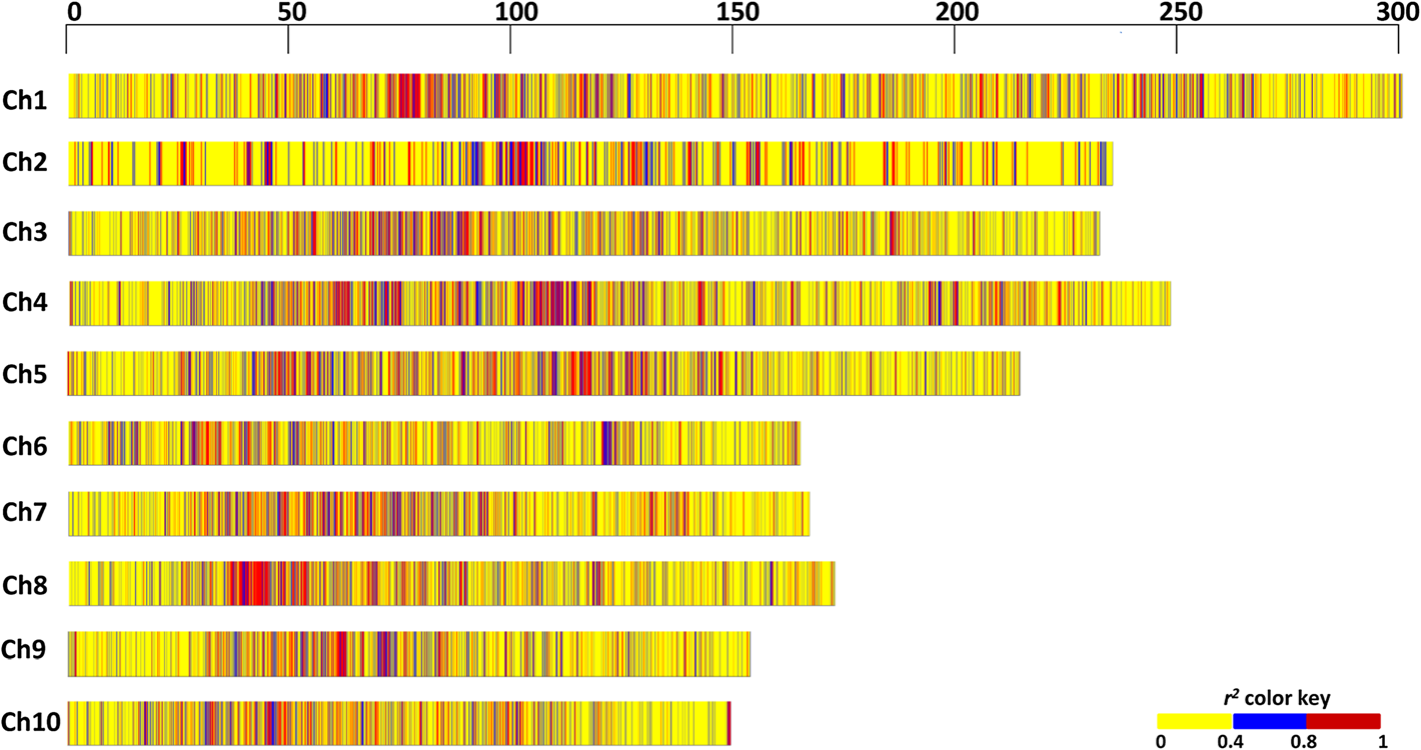
**The distribution of low (*r***^***2***^**≤ 0.4), intermediate (0.4 <*r***^***2***^**<0.8), and high (*r***^***2***^**≥ 0.8) pairwise LD regions along the chromosomes.** The pairwise LD pattern was measured using SNPs with a MAF of ≥0.05 across the maize association mapping panel. The length of the chromosomes is shown in Mb.

We characterized genome-wide LD in subtropical elite breeding lines and found several low to high LD regions within and across chromosomes (Figure 7). Approximately 11% of SNPs with high LD (*r*^*2*^ ≥ 0.8) were scattered throughout the genome. The high LD regions were mostly interspersed with low LD regions, indicative of maize genome complexity and the random nature of recombination events across the genome [15,53]. However, extensive regions of high LD were found on chromosomes 3 and 8 from 94.6–95.1 Mb and 48.7–51.7 Mb, respectively; their presence may be due to recent allelic drift in the population (Figure 7) [54]. In almost all chromosomes, LD was lower near telomeric regions and higher in centromeric and pericentromeric regions. Low LD regions may be rich in functional genes and actively involved in recombination [15,53].

On the other hand, high LD regions were distributed uniformly along the chromosomes; this indicates that these loci were single or multiple genes of agronomic importance that were selected for by a number of breeding programs, thereby creating LD between linked and unlinked loci over time [55]. These regions may also be a consequence of several other factors, including low recombination rates [56], selective sweeps [11,17,57], population bottlenecks [53], directional selection for specific traits [58,59], and ascertainment bias [60].

**Figure 8:**
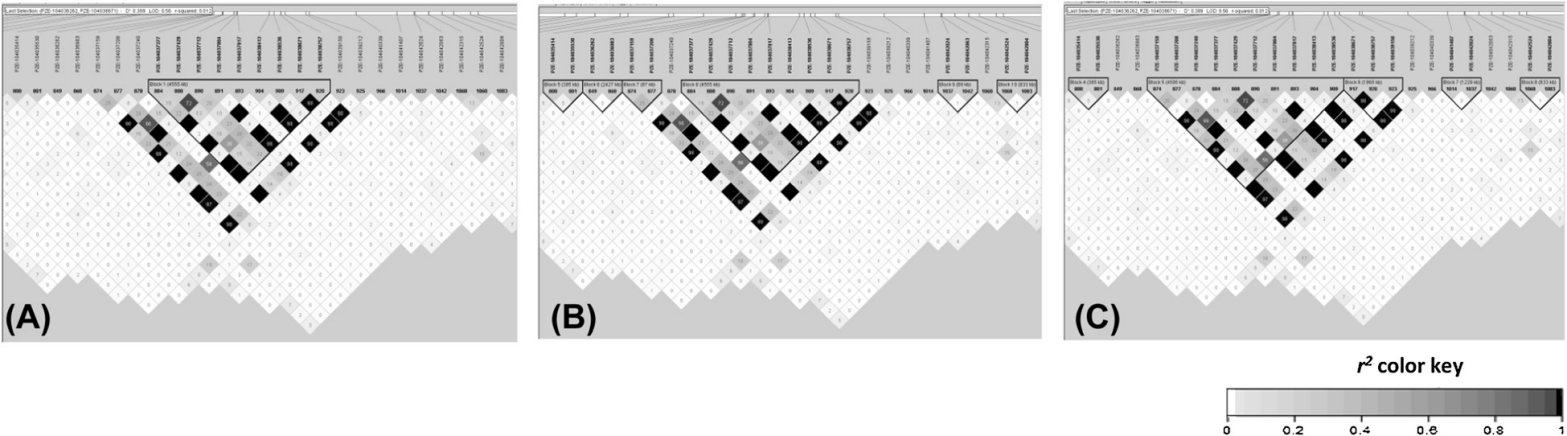
**Haplotype blocks ranging from 37.41 Mb to 77.67 Mb on chromosome 4 as visualized in a 20-Mb sliding window on Haploview.A)** Confidence intervals model, **B)** Four gamete rule and **C)** Solid spine of LD model. The inverted triangle represents haplotype blocks.

Haplotypes are a function of population size, genetic diversity, and the extent of LD. The use of a large number of SNPs would increase their coverage, since most of the genomic variation would then be available for analysis. In the present study, genome-wide SNP genotyping revealed a total of 252 haplotype blocks varying in size from <1 Kb (2 SNPs) to 15.8 Mb (262 SNPs). The CI model identified fewer and shorter haplotype blocks than FGR and SS models (15.8 Mb), however; this difference may be due to blocks with strong LD in a high-confidence bound cut-off in the former model (Figure 8). These haplotype blocks are indicative of the magnitude of recombination across the genome and imply the selection of their corresponding alleles. Interestingly, there were more haplotype blocks, suggesting fixation of alleles [61], on chromosome 3. Many long terminal repeats or retrotransposons [62], which are not uncommon in maize [63], were also present. These latter regions are considered to be gene poor [64] and do not normally undergo recombination; they are thus highly conserved in a population.

When performing association mapping, an understanding of the LD decay pattern is important, because mapping resolution is correlated with LD decay [51]. A low LD population will facilitate high-resolution gene mapping [15,16], whereas a high LD population will only allow for coarse mapping [9]. In our study, LD decay distance was found to be 200–300 Kb, comparable to that of European elite breeding lines (*r*^*2*^ = 0.1 at ~500 kb) [9]. Based on the slow decay pattern, the population of elite breeding lines had obviously undergone several rounds of selection for favorable traits. Previous studies have revealed that when LD decays at less than 10 Kb, the population is highly genetically diverse [12,13], possibly as a consequence of inter-breeding, selection, population bottlenecks, geographical isolation [65], genetic drift [54], and population structure [13]. LD declines rapidly (e.g., *r*^2^ ≤ 0.1 within 1,500 bp) in various maize lines [13,16]. In our population, LD decay was more rapid at *r*^*2*^ ≤ 0.2 (5–10 Kb) than at *r*^*2*^ ≤ 0.1 (200–300 Kb), but we can assume that our panel still offers good resolution for gene mapping.

The removal of SNPs with a MAF < 0.05 facilitates high-power gene mapping of a population, as the inclusion of minor alleles may lead to inaccurate LD estimation. To analyze LD decay patterns at different MAF cut-off levels, we measured LD using SNPs with 0%, ≥ 5%, and ≥ 10% MAF cut-off levels. A change in the LD decay pattern was noticed between 0% and 5% cut-off levels, whereas an increase in the MAF threshold from 5% to 10% did not markedly affect the mean *r*^*2*^ across the 10 chromosomes. This implies that the allele frequency did not change drastically at the MAF ≥ 5% level, thereby increasing the frequency of common alleles. Another explanation for this result could be the occurrence of a domestication bottleneck leading to the elimination of rare alleles and hence shifting allele frequencies towards intermediate values [66]. Rare alleles may have become fixed in the population during selection for agronomic traits. It should be noted, however, that high frequency markers are required to detect all rare alleles in a population [67]. In addition, founder lines used for creating SNP chips may not exhibit the whole gamut of allelic diversity of a species owing to ascertainment bias [68]. Furthermore, small sample sizes may cause alleles to be underrepresented on SNP arrays [29], further limiting the detection of rare and minor alleles.

### Population structure

The presence of structure in a selected population is due to various processes, such as population bottlenecking, genetic drift, and selection. Non-genetic factors, including genotyping error [47] and ascertainment bias [68], also contribute to population structure. Using ADMIXTURE, we identified two major and two minor subsets in our population. This result suggested the presence of unequal allele frequencies in the population, which might be due to non-random mating among individuals [65].

Indian maize breeding programs use both yellow and white lines, and the generation of lines derived by crossing these types is frequently carried out to maintain quality. In our study, few white lines appeared in groups containing mostly yellow lines, indicating the eventual outcome of inter-mating. CMLs developed by CIMMYT (http://www.seedsofdiscovery.org) have also been used in several breeding programs in India. These lines have been selected based on adaptability as well as specific traits. Hence, CML lines integral to the Indian breeding program were included in our analysis along with the already adapted Indian lines.

The population structure uncovered by ADMIXTURE was congruent with the distribution pattern identified by PCA. The PCA-based genotype distribution clearly showed two subsets covering more than 87% of the genotypes. Two of the subgroups from the ADMIXTURE analysis were not wholly supported by PCA, however, as some of the genotypes from these two minor clusters of ADMIXTURE were mixed with the major PCA groups. The similar grouping of subsets from ADMIXTURE and PCA implies that these results may be used to correct for population structure for association mapping [69]. In contrast, overall results from ADMIXTURE and genetic distance matrix analyses were not comparable, similar to the findings of an earlier study [31].

### Genetic diversity

We assessed the genetic diversity of the 240 subtropical maize lines with the aim of developing heterotic pools for Indian breeding programs. Numerous selfing generations in elite breeding lines can lead to a reduction in harmful alleles [70]. In such cases, a heterotic pool containing the resulting genotypes has the potential to increase hybrid vigor. Further understanding of their genetic diversity would be useful for making selective crosses among the lines to maximize genotype heterotic potential.

Genetic variability has been studied previously using SSRs and SNPs [29,31,32]. Mean genetic dissimilarity (0.36) in the present study was considerable given the number of SNPs used, and was comparable to values from previous genetic assessment studies [29-31]. NJ analysis of genetic dissimilarity coefficients separated the population into two major groups and one minor group. The distribution of genotypes provided ample options for choosing different parental combinations for a hybrid development program (Additional file 10: Table S4). Genotypes belonging to early, medium, and late maturity groups fell into different clusters. These genotypes were variously tolerant to abiotic stresses, resistant to diseases, or possessed other special characteristics (http://www.maizeindia.org). Our study thus provides information for developing new hybrids possessing different maturity-trait combinations by performing selective crosses between and within maturity groups based on genetic distances.

Several parental pairs with high genetic dissimilarity were identified. Yellow lines NAI 147 (Group A) and CML 69 (Group B) from the late maturity group had a high dissimilarity coefficient (0.43). NAI 147 was also very dissimilar (0.43) to CML 193 from the medium maturity group. The genetic distance between such distant lines suggests that their crosses would show good heterosis. The selection of parental pairs based on genetic dissimilarity would be a good starting point to identify potential heterotic combinations. Before exploiting parental pairs in heterosis breeding programs, however, their agronomic traits should first be tested for combining ability.

Most of the CML-derived lines in our study clustered together with remaining Indian lines into groups 2A, 2B, and 3A. Because the CML lines are resistant to several diseases (http://www.seedsofdiscovery.org), hybridization of CML lines with other lines would be desirable to impart disease resistance and to realize their heterotic potential. Genetically dissimilar, stress-tolerant parents can also be used for the development of QTL mapping populations for target traits. Biparental populations developed from individuals with contrasting traits, selected from within the association mapping panel, can serve as association mapping validation tools.

### Prospects for genome-wide association studies (GWAS)

Our association mapping panel of Indian breeding lines, the most saturated panel currently reported with respect to marker density, not only contributes to an understanding of their genetic architecture, but also helps elucidate LD and population structure and may be useful for GWAS. The distribution of high and low LD regions across the genome provided an opportunity to identify target genes of agronomic interest. Haplotype blocks identified in the genome, such as the 74 blocks on chromosome 2, can be exploited for GWAS. Slow LD decay was observed, however, enabling only coarse mapping at a resolution of 200–300 Kb. Even at coarse resolution, it would still be possible with the help of *in silico* tools and maize gene prediction models (http://www.maizesequence.org) to identify putative genes for target traits. On the other hand, we observed very rapid LD decay across chromosomes when the cut-off was shifted from *r*^*2*^ ≤ 0.1 to 0.2. Consequently, the fine mapping potential of our subtropical maize panel should not be ignored. Our analysis uncovered strong population structure, which limits this panel’s use for GWAS; however, the structure could be corrected for through the use of statistical models based on ADMIXTURE and PCA results. We believe that our association mapping panel with genome-wide SNPs will provide an opportunity to map genes of agronomic importance.

## Conclusions

We characterized subtropical elite maize breeding lines using a large number of high-quality SNPs. Assessment of marker-trait associations is facilitated by the availability of saturated SNPs across the genome. Genomes of these maize lines were found to have both low and high LD regions. The slow LD decay observed in the population was attributed to the inclusion of elite breeding lines in this study. Congruency between the ADMIXTURE and PCA results increases the confidence that the population structure can be corrected for during association mapping. The genetic diversity uncovered in the assayed population can be used to develop heterotic pools for exploitation of elite breeding line hybrid vigor.

### Availability of supporting data

The raw SNP data (Submission # https://10.6070/H4BG2KX8) has been submitted to the website: http://www.labarchives.com/.

## Abbreviations

CI: Confidence interval; CS: Cluster separation; FGR: Four gamete rule; GD: Genetic diversity; GT: GenTrain; GWAS: Genome-wide association studies; Kb: Kilobase; LD: Linkage disequilibrium; MAF: Minor allelic frequency; Mb: Megabase; NJ: Neighbor-joining; PCA: Principal component analysis; PC: Principal components; PIC: Polymorphism information content; SNP: Single nucleotide polymorphism; SS: Solid spine of LD.

## Competing interests

The authors declare that they have no competing interests.

## Authors’ contributions

NT and HSG conceived and designed the experiments; FH, KS, SMl, SMn and PMN performed the experiments; NT, SMl, AR, RS, KA and TS analyzed the data; AMSV and TM helped in data generation; and NT, KA and SMn wrote the paper. HSG coordinated the research. All authors read and approved the final manuscript.

## Supplementary Material

Additional file 1: Table S1 Attributes of genotypes used in the genotyping panel. Maturity groups: E, early; M, medium; L, late. Kernel color: Y, yellow; W, white; SD: Semi-dent; SF: Semi-Flint. Click here for file

Additional file 2: Figure S1 GenTrain (GT) and cluster separation (CS) scores for 56,110 SNPs. Each SNP had an individual GT and CS score across the subtropical panel. Click here for file

Additional file 3: Figure S2 SNP coverage across all chromosomes. The average number of SNPs/chromosome across the whole genome was 2962. Click here for file

Additional file 4: Figure S3 Characteristics of 29,619 high-quality SNPs. Gene diversity (GD), polymorphic information content (PIC), and minor allelic frequency (MAF) averaged for 240 individuals. Click here for file

Additional file 5: Table S2 Characteristics of haplotype blocks obtained from three different models using 10-Mb and 20-Mb windows. Click here for file

Additional file 6: Figure S4 Effects of minor allelic frequency (MAF) on LD decay. Comparison of mean *r*^*2*^ values at MAF levels of 0%, ≥ 5%, and ≥ 10% across the subtropical panel. Click here for file

Additional file 7: Figure S5 Graphical representation of genotype grouping based on allele frequency at different K levels. Each of the 240 genotypes is represented by vertical lines partitioned into the respective clusters denoted by K (range, 2–7). Click here for file

Additional file 8: Table S3 Membership value of the genotypes of the association mapping panel generated by ADMIXTURE. Click here for file

Additional file 9: Figure S6 Genetic dissimilarity coefficient of all pairwise genotypes. The genetic dissimilarity matrix was calculated between 240 individuals using 29,619 SNPs and Roger’s modified distance. Click here for file

Additional file 10: Table S4 Genotype pairs selected on the basis of genetic dissimilarity (> 0.35) and other traits for various breeding purposes. Note: Maturity groups: E, early; M, medium; L, late. Kernel color: Y, yellow; W, white. Click here for file
