# Supplementary figures and images for: Unraveling the genetic architecture of subtropical maize (Zea mays L.) lines to assess their utility in breeding programs
Source: BMC Genomics. 2013 Dec 13;14:877. doi: 10.1186/1471-2164-14-877 (PMC3867671; doi:10.1186/1471-2164-14-877)

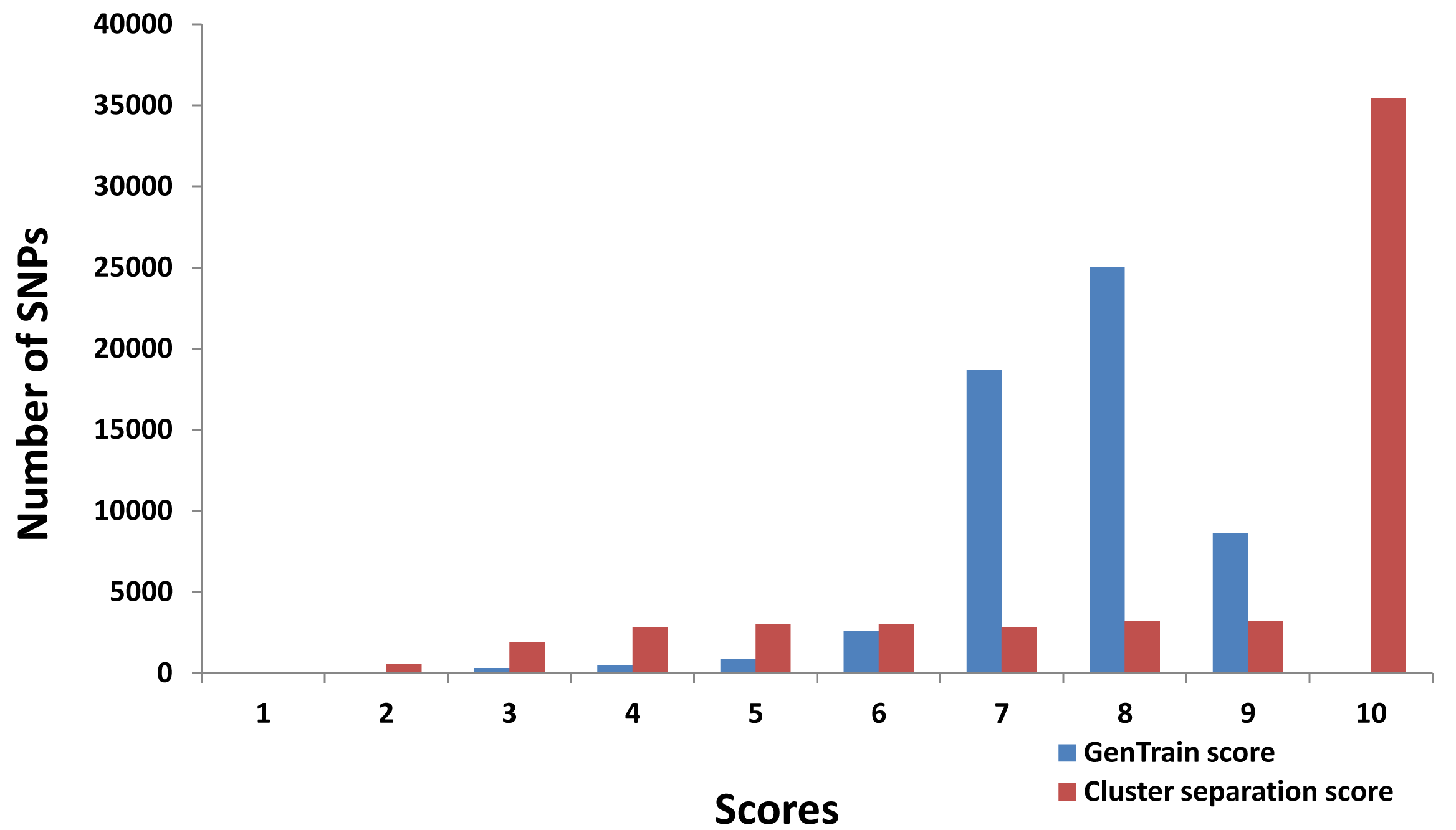

Supplement: Additional file 2: Figure S1 — GenTrain (GT) and cluster separation (CS) scores for 56,110 SNPs. Each SNP had an individual GT and CS score across the subtropical panel. [file 1471-2164-14-877-S2.tiff]

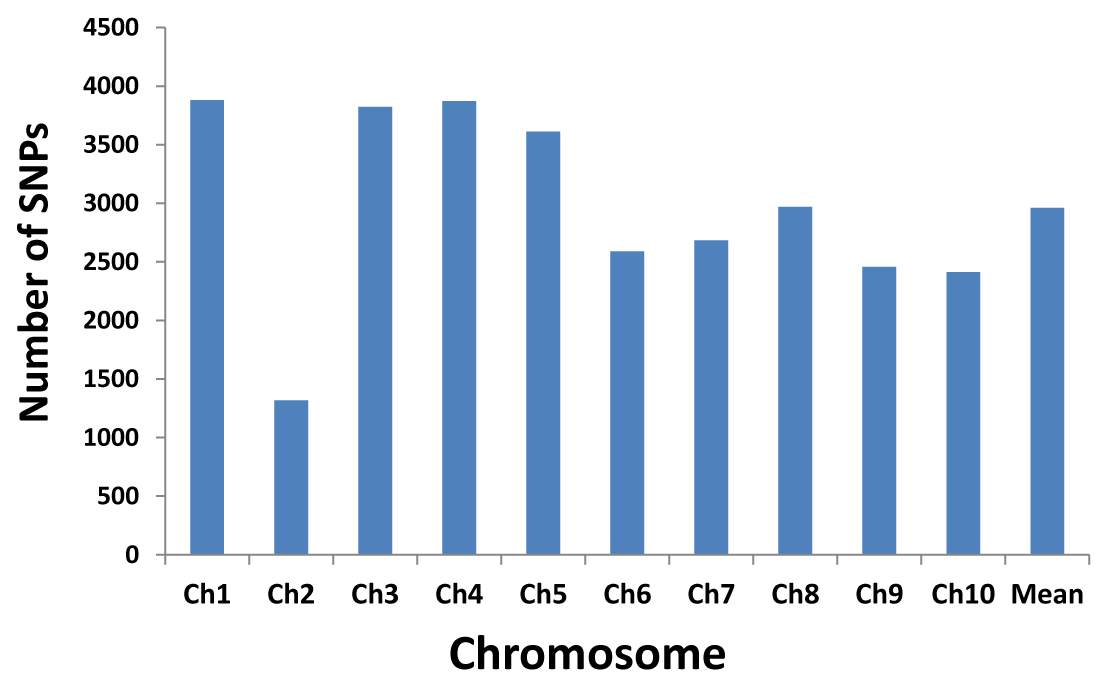

Supplement: Additional file 3: Figure S2 — SNP coverage across all chromosomes. The average number of SNPs/chromosome across the whole genome was 2962. [file 1471-2164-14-877-S3.tiff]

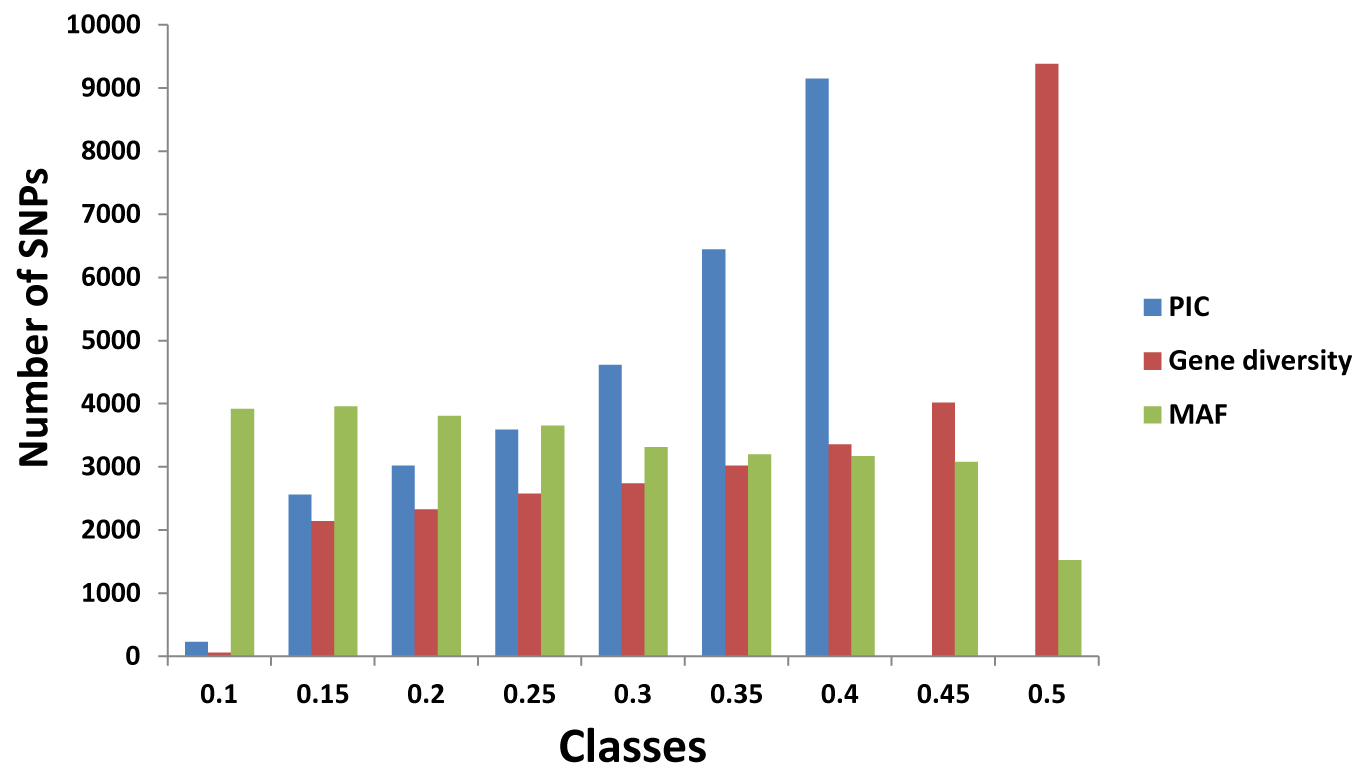

Supplement: Additional file 4: Figure S3 — Characteristics of 29,619 high-quality SNPs. Gene diversity (GD), polymorphic information content (PIC), and minor allelic frequency (MAF) averaged for 240 individuals. [file 1471-2164-14-877-S4.tiff]

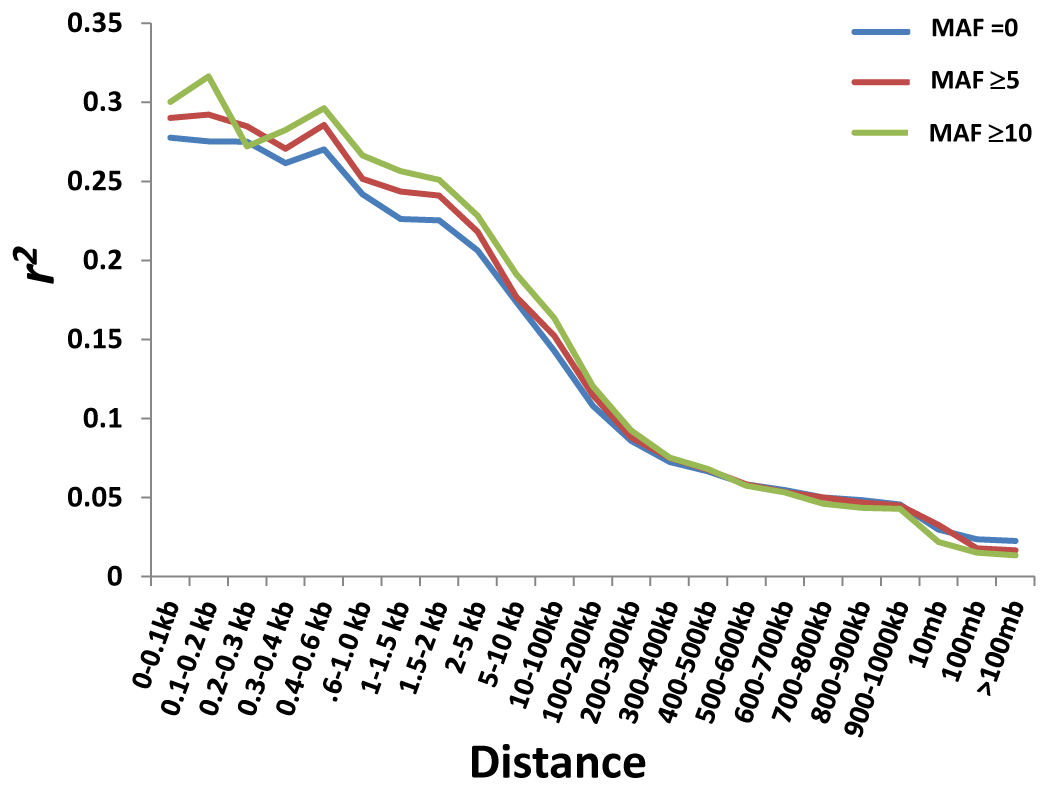

Supplement: Additional file 6: Figure S4 — Effects of minor allelic frequency (MAF) on LD decay. Comparison of mean r 2 values at MAF levels of 0%, ≥ 5%, and ≥ 10% across the subtropical panel. [file 1471-2164-14-877-S6.tiff]

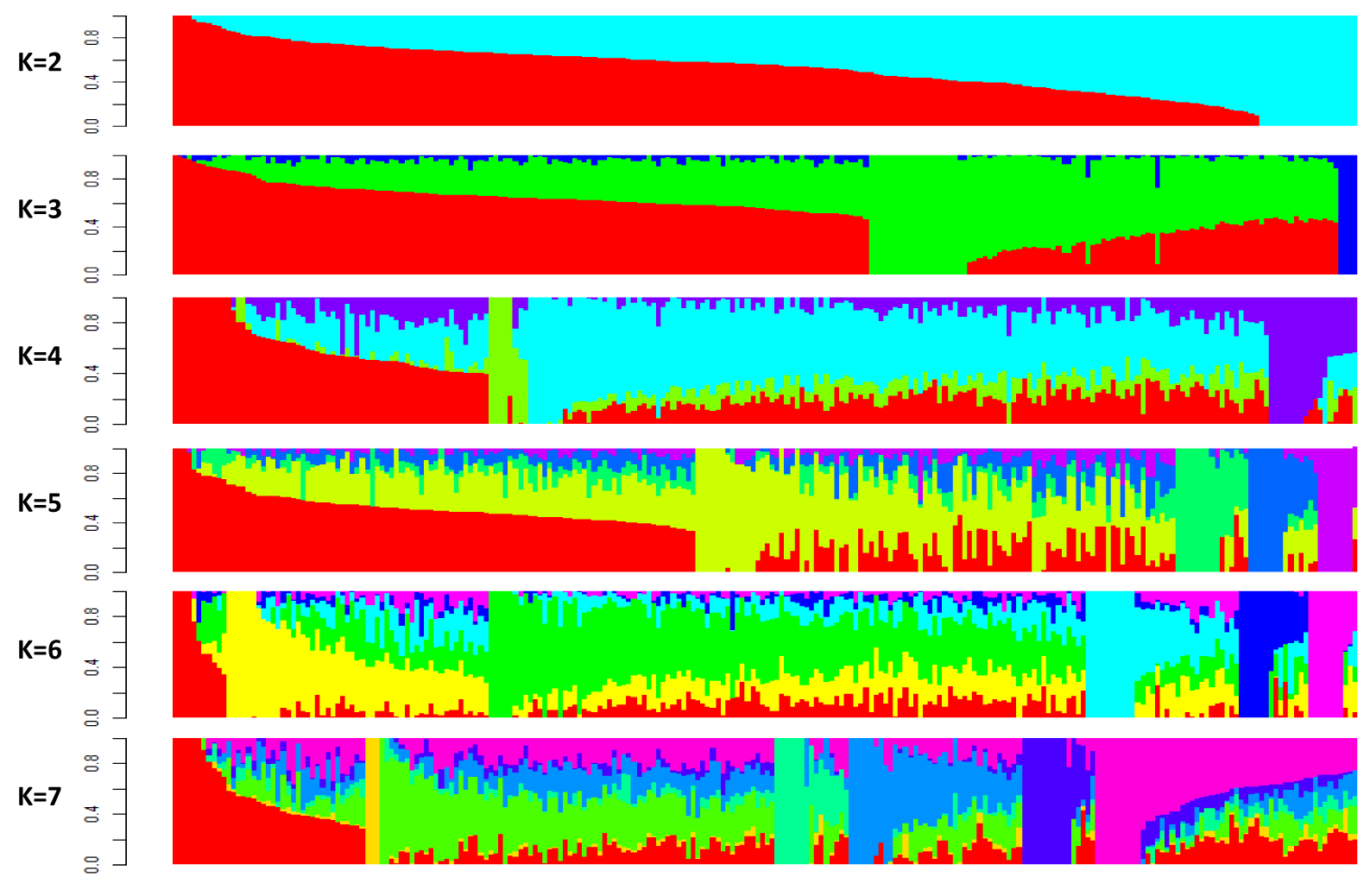

Supplement: Additional file 7: Figure S5 — Graphical representation of genotype grouping based on allele frequency at different K levels. Each of the 240 genotypes is represented by vertical lines partitioned into the respective clusters denoted by K (range, 2–7). [file 1471-2164-14-877-S7.tiff]

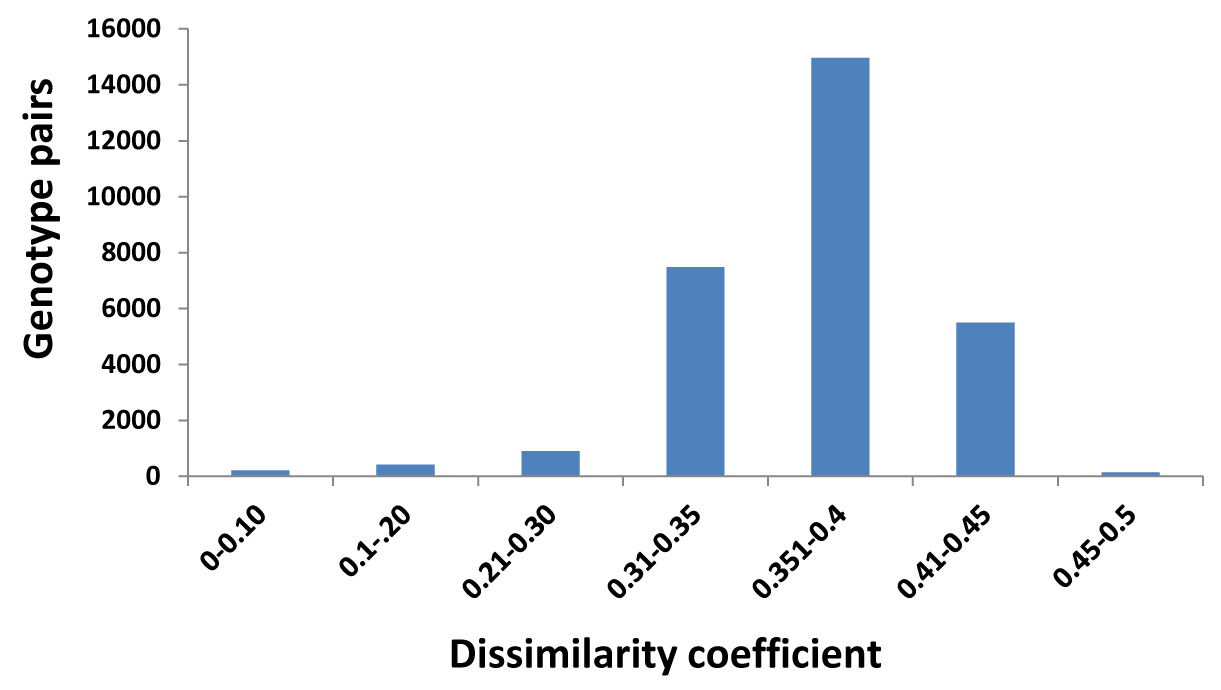

Supplement: Additional file 9: Figure S6 — Genetic dissimilarity coefficient of all pairwise genotypes. The genetic dissimilarity matrix was calculated between 240 individuals using 29,619 SNPs and Roger’s modified distance. [file 1471-2164-14-877-S9.tiff]
