# Supplementary material for: Unraveling the genetic architecture of subtropical maize (Zea mays L.) lines to assess their utility in breeding programs
Source: BMC Genomics. 2013 Dec 13;14:877. doi: 10.1186/1471-2164-14-877 (PMC3867671; doi:10.1186/1471-2164-14-877)
Supplement: Additional file 5: Table S2 — Characteristics of haplotype blocks obtained from three different models using 10-Mb and 20-Mb windows. [file 1471-2164-14-877-S5.docx]

**Additional file 8: Table S2 Characteristics of haplotype blocks obtained from three different models using 10-Mb and 20-Mb windows.**

| **Chromosome** | | **Confidence interval** | | **Four gamete rule** | | **Solid spine of LD** | |
| --- | --- | --- | --- | --- | --- | --- | --- |
|  |  | **Haplotype blocks (number)** | **Average length of haplotype**  **block (Kb)*** | **Haplotype blocks (number)** | **Average length of haplotype block (Kb)*** | **Haplotype blocks (number)** | **Average length of haplotype block (Kb)*** |
| 1 | | 0 | - | 23^10, 20^ | 1594.8 (55–7811)^10, 20^ | 23^10, 20^ | 1592.3 (55–7836)^10, 20^ |
| 2 | | 6 | 27.0 (1–74)^10^ | 74^10, 20^ | 1520.4 (1–9680)^10, 20^ | 72^10^ | 1827.2 (1–9559)^10^ |
|  |  |  | 27.0 (15–74)^20^ |  |  | 68^20^ | 2133.5 (1–13865)^20^ |
| 3 | | 1 | 1.0^10, 20^ | 20^10, 20^ | 1559.9 (1–7688)^10^ | 20^10^ | 1771.6 (1–7522)^10^ |
|  |  |  |  |  | 2674.1 (1–15845)^20^ | 21^20^ | 2825.0 (1–15845)^20^ |
| 4 | | 2 | 2287.0 (19–4555)^10, 20^ | 31^10, 20^ | 1267.2 (16–4555)^10, 20^ | 27^10, 20^ | 1919.3 (16–254)^10^ |
|  |  |  |  |  |  |  | 1741.6 (16–8254)^20^ |
| 5 | | 2 | 1524.5 (5–3044)^10, 20^ | 16^10, 20^ | 1719.1 (5–4767)^10, 20^ | 19^10, 20^ | 1141.1 (3–4937)^10, 20^ |
| 6 | | 3 | 4.6 (2–7)^10, 20^ | 21^10, 20^ | 1589.9 (5–8322)^10, 20^ | 20^10^ | 1890.7 (5–8322)^10^ |
|  |  |  |  |  |  | 19^20^ | 2175.5 (5–10820)^20^ |
| 7 | | 3 | 1150.0 (4–3433)^10, 20^ | 17^10, 20^ | 875.0 (4–3719)^10, 20^ | 20^10, 20^ | 1025.0 (4–3433)^10, 20^ |
| 8 | | 0 | - | 17^10, 20^ | 1244.5 (65–4491)^10, 20^ | 19^10, 20^ | 1219.6 (65–7088)^10, 20^ |
| 9 | | 0 | - | 14^10^ | 1142.9 (114–6864)^10^ | 11^10, 20^ | 711.7 (3–2787)^10, 20^ |
|  |  |  |  | 13^20^ | 1198.7 (114–6864)^10, 20^ |  |  |
| 10 | | 1 | 8.0^10, 20^ | 20^10, 20^ | 1196.4 (8–4712)^10, 20^ | 21^10, 20^ | 957.3 (8–2404)^10, 20^ |
| **Total** | **18** | |  | **253^10^** |  | **252^10^** |  |
|  |  |  |  | **252^20^** |  | **248^20^** |  |
| **Mean** |  | | **714.6^10, 20^** |  | **1371.0^10^** |  | **1405.6^10^** |
|  |  |  |  |  | **1488.0^20^** |  | **1552.3^20^** |

* Values in parenthesis indicated minimum and maximum block size (in Kb).

^10^ Unique to 10-Mb window; ^20^ Unique to 20-Mb window; ^10, 20^ Common to 10- and 20-Mb windows.
